# Fertility-sparing treatment for patients with endometrial carcinoma

**DOI:** 10.52054/FVVO.15.1.061

**Published:** 2023-03-31

**Authors:** T Ind

**Affiliations:** Head of Gynaecological Oncology, Royal Marsden Hospital, London, UK

We are seeing an increasing number of collaborations between professional organisations which have a mutual area of interest for the provision of guidance to healthcare professionals. These collaborations probably have several benefits at multiple levels; they not only reduce cost of production by sharing it but more importantly they bring expertise and knowledge together to explore the most appropriate management options and push the boundaries to the limit. We now have a joint guideline from the European Society of Gynaecological Oncology (ESGO), European Society of Human Reproduction and Embryology and (ESHRE) and European Society for Gynaecological Endoscopy (ESGE) for the fertility-sparing treatment of patients with endometrial cancer simultaneously published in three journals (Rodolakis et al., 2023a; Rodolakis et al., 2023b; Rodolakis et al., 2023c). ESGO has also addressed this topic in their recent guideline for the management of uterine cancer in conjunction with European Society for Radiotherapy & Oncology (ESTRO) and European Society of Pathology (ESP) (also published in three places) (Concin et al., 2021a; Concin et al., 2021b; Concin et al., 2021c). For a gynaecological oncologist practicing in the UK this competes with the British Gynaecological Cancer Society (BGCS) guidelines (Morrison et al., 2022) and comes in parallel with an update in the American National Comprehensive Cancer Network (NCCN) guidelines (Abu-Rustum et al., 2021).

In 1985 the incidence of endometrial cancer in the UK was 13.5 per 100,000. In 2018 this had doubled to 30 per 100,000 making uterine cancer the most common gynaecological malignancy in the UK (Cancer Research UK., 2019). The incline in the incidence matches the increase in levels of obesity. This trend seems to be mirrored across Europe and the rest of the western world. There are now nearly 10,000 new cases of endometrial cancer a year in the UK. However, the peak incidence is in the 75 – 79-year-old age group with only about 150 women (about 1.5%) developing endometrial cancer in the UK before the age of 40. The majority of women under 40 years of age with endometrial cancer will either have a malignancy unsuitable for fertility-sparing treatment or will have completed their family already as most are 35 years old and over. Even in the largest centres serving populations of nearly two million women, there is unlikely to be more than one or two such cases a year. Having an up-to-date evidenced base guideline is extremely useful for the clinician for when they encounter a case as it saves them from trawling the literature to advise on management.

There are some particularly good aspects of this guideline. One of these is the understanding of the need to respect a woman’s perception of the relevant importance of different outcomes (i.e., oncological versus fertility). This is often ignored in recommendations for fertility-sparing surgery in cervical cancer where the oncology outcome is seen to trump the desire a woman has to preserve her fertility (Cibula et al., 2018a; Cibula et al., 2018b; Cibula et al., 2018c). This is an important point that I repeatedly stressed, in relation to cervical cancer treatments, when drafting a similar section for the BGCS guidelines for cervical cancer (Reed et al., 2021; Ind, 2021). The importance of the need to involve fertility specialists and to give some form of qualitive information on reproductive potential is both clearly important and perhaps lacking in other guidelines. Another aspect to promote this guideline is the structured way in which the evidence has been collected and excellent evidence base supporting the recommendations given. Finally, the mature discussion regarding the approach to women with Lynch syndrome is something to contemplate and clearly a focus for research in the future.

There are some aspects that I think could be improved on. One of these is the lack of discussion about obstetric outcomes after fertility-sparing treatment. Conception and fecundity are not the only pregnancy outcomes that need to be discussed as part of an informed consent process.

There are two contradictory statements in the guideline. In one recommendation it states “Fertility-sparing treatment is considered for endometrioid patients with endometrial carcinoma with grade 1, stage 1a without myometrial invasion and without risk factors” yet in another it states, “If an early and focal myometrial invasion (1-2mm) is suspected from the resection material a fertility-sparing approach may be discussed on a case-by-case basis”. Are you allowed to offer this approach to a woman with myometrial invasion or not? If a woman declines hysterectomy, surely a doctor can give her another treatment even if it is not the most effective.

There is another contradiction in relation to the medical management of endometrial cancer. It states “Medroxyprogesterone acetate (400–600mg/day) or megestrol acetate (160–320mg/day) is the recommended treatment. Treatment with levonorgestrel intrauterine device in combination with oral progestins with or without gonadotropin- releasing hormone analogues can also be considered (IV, B)”. This contrasts to their other aligned guideline published in this journal (Concin et al., 2021a) where they say “A levonorgestrel-intra-uterine device at a dose of 52mg, alone or in combination with oral progestins, is a safe and effective approach (Level of evidence III, grade B)”. These two statements differ in their recommendation for an intrauterine system alone without the need for additional systemic hormone therapy.

Despite these minor issues, this guideline comes towards the top of my league table for recommended practices in gynaecological oncology. This is mainly because of the excellent evidence base on which it was formed, and the dilemmas overcome in interpreting evidence which can be of a low volume, a low quality or often completely absent.

The management of endometrial cancer has changed beyond recognition in only the last five years. It has evolved from the domain of the special interest gynaecologist to the sub-specialist team. The old behaviour of an open radical hysterectomy and pelvic lymphadenectomy has moved to laparoscopic surgery and robotics with sentinel lymph node dissection favoured over a full lymphadenectomy. The methods for selecting patients for different types of adjuvant treatment has become more precise. The new molecular profiling classification has helped to further define treatment by the recommendation for testing for p53 abnormalities, POLE staining, mismatch repair (MMR) staining & hypermethylation, and Lynch testing. In light of all these changes, the management of endometrial cancer should be re-analysed to some degree.

In my experience, fertility-sparing requests are often initiated by a patient who is not prepared to accept the finality of losing her womb. A woman’s desire to preserve fertility may trump everything else. Irrespective of guidelines a woman often choses her own route. Informed consent is then the responsibility of the doctor and this guideline provides the requisite information to inform that choice. The doctor then has a duty of care to that patient irrespective of her choice. Last week I met Henry (not his/her real name). He was six months old. His mother had a high body mass index and presented to me aged 45 with a grade 1 uterine cancer in the background of a multiple fibroid uterus and adenomyosis. She had no partner. In part due to the excellent skill of her fertility specialists and against my advice she conceived Henry who is a happy and loved boy with the world ahead of him. My fertility colleague described him as “a miracle baby”. Anecdote is not really evidence but something to ponder on. His mother has now consented to a hysterectomy.
